# Redefining the role of perfusionists in ECMO: from technical operators to clinical stakeholders

**DOI:** 10.1051/ject/2025046

**Published:** 2025-12-17

**Authors:** Salman Pervaiz Butt, Salman Abdulaziz, Nabeel Razzaq, Arun Kumar, Fazil Ashiq, Umer Darr, Gopal Bhatnagar

**Affiliations:** 1 Perfusion Services, Heart Vascular and Thoracic Institute, Cleveland Clinic Abu Dhabi PO Box 112412 Abu Dhabi United Arab Emirates; 2 Critical Care Services Administration, King Fahad Medical City Riyadh Saudi Arabia; 3 Cardiac Surgery, Heart Vascular and Thoracic Institute, Cleveland Clinic Abu Dhabi PO Box 112412 Abu Dhabi United Arab Emirates; 4 Anesthesiology Institute, Cleveland Clinic Abu Dhabi PO Box 112412 Abu Dhabi United Arab Emirates

**Keywords:** ECMO, Technical Operators, Clinical Stakeholders

## Abstract

Extracorporeal Membrane Oxygenation (ECMO) is increasingly used in refractory cardiac and respiratory failure, yet the role of perfusionists remains narrowly defined. While traditionally viewed as technical operators, perfusionists possess advanced expertise that are essential across the continuum of care. Their exclusion from key clinical and academic roles represents a structural gap possibly with implications for safety and outcomes. This letter advocates for redefining perfusionists as clinical stakeholders, formally integrated into decision-making, quality improvement, and research initiatives. Their engagement can enhance ECMO practice across a multitude of areas. Recognizing perfusionists as integral members of the multidisciplinary team is essential to advancing outcomes, and meeting the demands of increasingly complex extracorporeal therapies.

To the Editor

Extracorporeal Membrane Oxygenation (ECMO) has become a central therapeutic modality in the management of patients with refractory cardiac and respiratory failure. As the complexity of patient profiles and circuit technologies increases, the delivery of safe and effective ECMO support necessitates a well-integrated, multidisciplinary team. Despite the foundational role of clinical perfusionists in the initiation and technical operation of ECMO circuits, their exclusion from key phases of care, particularly in ongoing multidisciplinary discussions, protocol development, and research, is a persistent and under-addressed gap in contemporary ECMO programs [1].

Perfusionists are highly skilled professionals who routinely operate heart-lung machines and manage a wide range of extracorporeal support systems. Their expertise extends across several specialized areas, including hardware configuration, circuit design, blood conservation techniques, flow dynamics, membrane gas exchange, anticoagulation strategies, and device troubleshooting. They are also frequently involved in clinical education and training. Modern perfusionists are typically trained to at least a master’s degree level, acquiring in-depth knowledge of human physiology and cardiovascular pathology. Many also complete research projects as part of their MSc programs, which further enhance their academic and analytical capabilities.

Areas in which Perfusionists are involved with ECMO include operating rooms and intensive care units (ICUs). Perfusionists often serve as members of ECMO retrieval teams, responsible for the safe transfer of high-risk patients from referring hospitals. They also support patient rehabilitation by assisting with ECMO ambulation and physiotherapy sessions. Beyond the heart-lung machine, perfusionists manage a variety of mechanical circulatory support (MCS) devices, including intra-aortic balloon pumps (IABP), Impella, and long-term ventricular assist devices (VADs). These devices are often used in combination with ECMO, requiring an understanding of the complex interplay between systems to deliver optimal patient care [2].

Perfusionists’ competencies should not be limited to the technical setup of ECMO but extend across the full continuum of support. Critically, perfusionists can be engaged not only during ECMO initiation but also with the decision-making regarding candidacy, modality selection (e.g., VV vs. VA), hybrid configurations, and individualized flow goals. Their input could equally be essential in planning and executing weaning protocols and decannulation procedures, where circuit performance, patient physiology, and anticoagulation status must be assessed holistically. The absence of perfusion representation in these decisions can lead to avoidable complications, delayed interventions, or non-optimized strategies [3].

Furthermore, perfusionists are often excluded from daily ICU multidisciplinary rounds, where evolving patient dynamics are assessed and therapeutic directions set. Given that many complications during ECMO, such as oxygenator failure, increased transmembrane gradients, haemolysis, recirculation, or thrombus formation, are circuit-related, the omission of the team member most familiar with these variables represents a significant safety concern. Their routine involvement in rounds can enhance early detection of circuit-related issues, support timely clinical interventions, and ensure that the ECMO strategy remains technically feasible and physiologically sound [4].

Equally concerning is the limited participation of perfusionists in morbidity and mortality (M&M), multidisciplinary team meetings (MDTs), institutional audits, and quality improvement initiatives. Their absence removes a critical source of insight into ECMO system performance, device-related complications, and anticoagulation management errors, thus weakening the feedback mechanisms necessary for programmatic learning and evolution. ECMO is a technology-intensive therapy, and excluding the technical expert from process reviews not only impairs root-cause analyses but risks repetition of preventable adverse events [5].

Additionally, perfusionists remain underrepresented in ECMO-related research and academic authorship, despite being stewards of valuable circuit-level data. Their inclusion in clinical trials, registry contributions, and translational research efforts would enhance the methodological rigor and operational relevance of ECMO studies. Involving perfusionists in hypothesis generation, data interpretation, and dissemination of findings would support evidence-based refinements in circuit design, anticoagulation protocols, monitoring techniques, and safety standards.

The underrepresentation of perfusionists in critical phases of ECMO care is both a structural and cultural issue. It risks professional disengagement and missed opportunities for innovation, while perpetuating a fragmented approach to a therapy that demands interdisciplinary cohesion. To address these shortcomings, we strongly advocate for institutional frameworks and international guidelines that formally incorporate perfusionists into:

- ECMO initiation decision-making and configuration planning.
- Daily multidisciplinary ICU rounds and therapeutic reviews.
- Weaning assessments and structured decannulation protocols.
- M&M meetings, clinical audits, and quality assurance cycles.
- ECMO-focused research design, data collection, and academic publication.

The evolving complexity of ECMO therapy necessitates a redefinition of the perfusionist’s role from that of a technical operator to an integral clinical stakeholder. Their specialized knowledge in extracorporeal physiology, circuit mechanics, and anticoagulation management uniquely positions them to contribute meaningfully across the entire continuum of ECMO care. Formal integration of perfusionists into multidisciplinary clinical decision-making, daily ICU rounds, quality assurance initiatives, and research endeavours is essential to optimize patient outcomes, strengthen team dynamics, and uphold the standards of safe and evidence-based practice. As ECMO continues to advance in scope and sophistication, the inclusion of perfusionists in these domains must be recognized not as optional, but as a fundamental requirement (Figure 1).

**Figure 1:**
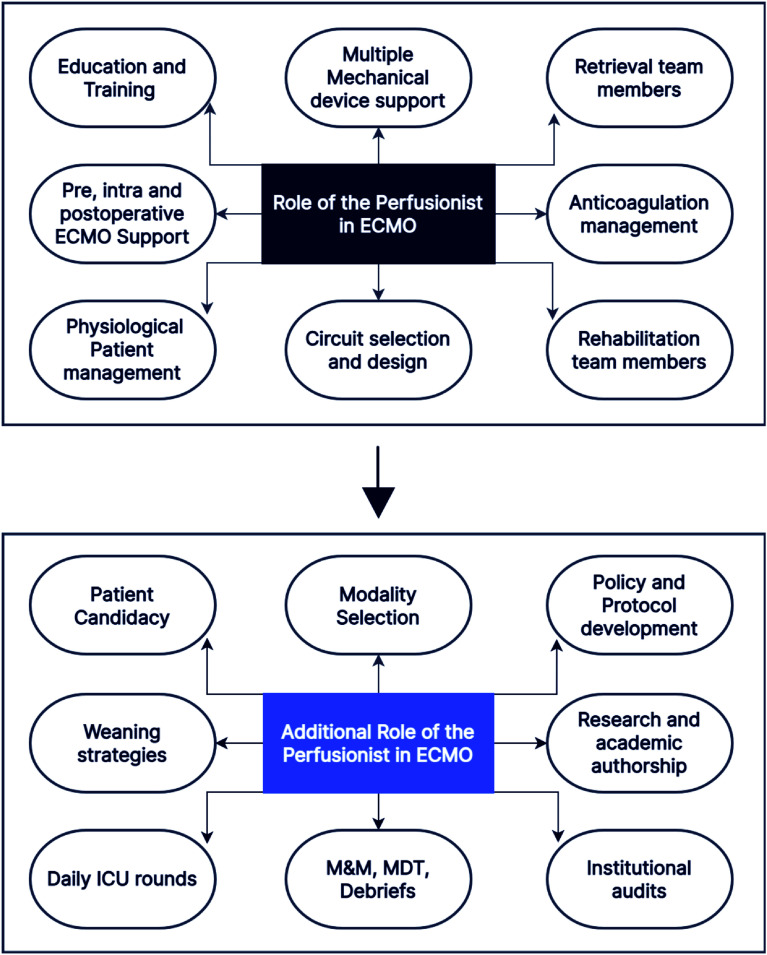
Two flowcharts illustrating the role of the perfusionist in ECMO. The first depicts the current scope of practice and areas of active involvement. The second presents potential expanded roles where perfusionist engagement could enhance workflow efficiency, support clinical decision-making, and improve patient outcomes.

## Data Availability

The data supporting this study’s findings are available from the corresponding author upon reasonable request.
